# Implementing a stakeholder-driven community diffusion-informed intervention to create healthier, more equitable systems: a community case study in Greenville County, South Carolina

**DOI:** 10.3389/fpubh.2023.1034611

**Published:** 2023-05-05

**Authors:** Larissa Calancie, Melissa L. Fair, Sally Wills, Kelsey Werner, Julia M. Appel, Travis R. Moore, Erin Hennessy, Christina D. Economos

**Affiliations:** ^1^ChildObesity180, Friedman School of Nutrition Science and Policy, Tuft’s University, Boston, MA, United States; ^2^Institute for the Advancement of Community Health, Furman University, Greenville, SC, United States; ^3^LiveWell Greenville, Greenville, SC, United States; ^4^Social System Design Lab, Washington University, St. Louis, MO, United States; ^5^School of Social Work, Boston College, Chestnut Hill, MA, United States; ^6^Department of Community Health, School of Arts and Sciences, Tuft’s University, Medford, MA, United States

**Keywords:** community coalition, systems change, childhood obesity, diffusion, systems dynamics, policy, systems and environmental changes, multi-sector stakeholders, group model building

## Abstract

This case study describes the application of a theory-informed, stakeholder-driven intervention with a group of 19 multi-sector stakeholders from an existing coalition to promote whole-of-community change that supports childhood obesity prevention. The intervention applied community-based system dynamics to design and implement activities that promoted insights into the systems driving childhood obesity prevalence and helped participants prioritize actions to influence those systems. This led to three new priority areas for the coalition: addressing food insecurity; building power among historically marginalized voices within the community; and supporting advocacy efforts to promote community-wide change beyond the coalition’s previous focus on organizational-level policy, systems and environment change. The intervention spurred the application of community-based system dynamics to other health issues and in partner organizations, which demonstrates paradigm shifts about how to address complex public health issues in the community.

## Introduction

Excess weight gain during childhood is a complex, serious public health issue. It increases the risk of obesity in adulthood and associated chronic diseases such as diabetes, hypertension, and cardiovascular disease (1). In Greenville County, South Carolina, surveillance data from youth in grades two and five indicates that 35.7% experience overweight or obesity, which is similar to national prevalence estimates (2, 3). Prevalence is disproportionately higher among Greenville’s African American (41.4%) and Hispanic youth (50.4%) (4). Equitably and sustainably reducing childhood overweight and obesity requires a comprehensive approach that addresses multiple drivers of excess weight gain simultaneously, including socioeconomic factors and issues of racism that systematically disadvantage communities of color (5, 6). Community coalitions are an approach for promoting equity and mobilizing childhood obesity efforts across sectors and organizations within a community (7, 8). Coalitions provide an arena for developing relationships, pooling resources, gaining knowledge and skills, identifying community needs and strengths, and empowering members as agents of change (9).

LiveWell Greenville (“LiveWell”) is a coalition that works to reduce childhood obesity in Greenville County, South Carolina, in addition to supporting healthy eating and active living for all age groups. The coalition has worked with over 250 organizations representing healthcare, schools, governments, parks/recreation, community members, and faith-based and social service organizations. LiveWell staff promote healthy eating and active living by convening partners to impact policy, systems and environmental (PSE) changes throughout Greenville County. Since 2010, LiveWell has actively shaped PSEs where children and their families live, learn, work, pray and play by leading organizations through assessments of current practices and supporting adoption of national best practices. For example, LiveWell worked with Greenville County Schools from 2011–2014 to transform the Greenville County School District’s food services’ school menus by transitioning from reliance on heavily processed meals to menus featuring scratch-made, nutritious meals that exceeded the national Healthy, Hunger Free Kids Act standards of 2010. The change influenced the food options available at lunch for more than 76,000 students in the district (10). The coalition also led an effort to expand a 22-mile countywide trail system and helped shift policies to promote healthy eating and physical activity opportunities in more than 100 organizations, including churches and private businesses, reaching tens of thousands of Greenville residents from 2011 to 2020 (11).

Despite significant success in engaging local businesses, early childhood centers, schools, afterschool programs, and congregations in organizational PSE change, LiveWell realized the coalition could not directly support all groups in Greenville County that wanted to implement organizational-level change. The staffing that would be required to provide organizational-level technical assistance to implement PSE changes that resulted in population-level impact was not possible or sustainable within the coalition’s budget and fundraising capabilities. In 2019, it became apparent that the coalition needed a new approach to create whole-of-community change. The coalition re-tooled its strategy to shape the community-level systems that facilitate or hinder organizational changes, making it easier for organizations to pursue PSE change without direct support from the coalition. In systems thinking terms, LiveWell wanted to create “conditions of systems change” and identify “high-leverage” systems change opportunities (12, 13). Of the six conditions of systems change that include policies, practices, resource flows, relationships and connections, power dynamics, and mental models, LiveWell was already adept at influencing policies, practices, and relationships (8, 9). In their next phase of work, coalition leaders and advisory board members wanted to strengthen their ability to promote resource and information flows, shift power dynamics, and change mental models to normalize decisions and values supporting equity and access to healthy, culturally meaningful foods and physical activity opportunities across the whole community. The coalition believed that doing so could introduce a paradigm shift away from commonly held beliefs placing responsibility for health outcomes on individuals toward ones that posit that interconnected systems (e.g., social, economic, food, healthcare, education) strongly influence health outcomes, and that those systems could be shifted to promote health for everyone in the Greenville community.

To help achieve this shift, LiveWell partnered with ChildObesity180, a research group at Tufts University, and an expert in community-based system dynamics, on the Catalyzing Communities initiative in Greenville. Catalyzing Communities is a whole-of-community approach to decreasing obesity prevalence, improving health, and promoting health equity in communities around the country. Whole-of-community approaches for obesity prevention are multilevel in that they operate within multiple levels of the social ecological model (e.g., intervention targeting environments to effect individual-level behaviors) and multi-component, in that they employ more than one strategy to affect change (14). The Catalyzing Communities project that includes the partnership with LiveWell is guided by a theory called Stakeholder-driven Community Diffusion (SDCD) (15, 16) which focuses on the work of a multi-sector group of stakeholders and integrates systems science methods including community-based system dynamics (CBSD), agent-based modeling, and social network analysis, to guide and evaluate the group’s work. This case study focuses on the use of CBSD in Catalyzing Communities; the use of an agent-based model and social network analysis is discussed in other publications (17). The theory hypothesizes that knowledge of and engagement with childhood obesity prevention activities can be increased “upstream” (i.e., within the stakeholder group) and diffused through social network connections to set the conditions for “midstream” PSE change, which ultimately is hypothesized to result in “downstream” outcomes at the individual level, like improved child health (18). The SDCD framework informed a three-phased intervention that has been implemented in three different communities (19, 20).

This case study describes the process of implementing an SDCD-informed intervention with LiveWell, reports how the intervention influenced systems in the county, and compares the SDCD intervention to LiveWell’s previous approach to obesity prevention. The study illustrates an approach for working with a coalition to promote whole-of-community change and considers how it could be adapted and replicated in other communities.

## Context

Greenville County covers 785 square miles, is one of the fastest growing counties in South Carolina and is the most populous. The county features rural farmland in the south and an urban city center with pockets of wealth and poverty. In 2021 the estimated county population was 523,542 and 22.9% of residents are under 18 years of age; 76.3% are white; 18.4% are African American; 9.5% are Hispanic (21). The median household income for 2015–2019 was $60,351, with 10.7% of the population living below the federal poverty level (22). In 2021, 42,980 individuals from 19,979 households participated in SNAP benefits (23). Out of the 92,584 total Full Benefit Medicaid members for Greenville County in 2019, 65% were children ages 0–18 and 28% adults ages 19–64 (24).

## Intervention elements

In the SDCD-informed intervention implemented with LiveWell, two community partners or “changemakers” (SW, MF) worked closely with the research team (LC, KW, JA, TRM, EH, CDE) from ChildObesity180 and an expert in CBSD to co-design intervention activities. The intervention incorporated CBSD, which uses group model building to engage participants in understanding how a system produces trends over time through structured activities and the use of graphs, models, and other visuals (25). The participatory activities use system dynamics conventions to illustrate feedback mechanisms that create system behavior (26, 27). CBSD also emphasizes building capabilities through participation to empower communities to use the approach to understand and change systems (28).

The two LiveWell changemakers attended a CBSD training hosted by the research team in March 2019, then, with assistance from the research team, began identifying multi-sector stakeholders who could participate in the intervention in September 2019. To select participating organizations and sectors, the coalition first identified the sectors within the county that had a significant influence on youth obesity (e.g., school system, health system, local government, philanthropy, community representative). Once the sectors and corresponding influencing organizations were considered, individuals perceived to have a high ability to influence resources and decisions related to child obesity prevention activities within their individual organizations and the county were recruited to participate. Nineteen people from ten sectors (e.g., healthcare, local government, philanthropy) agreed to participate in monthly meetings from October 2019 through January 2020, and then again in June and July 2020. Two individuals declined to participate. They generally held leadership roles in their organizations, with job titles such as executive director, pastor, health director, community member, or program officer. The group of convened stakeholders called themselves the LiveWell Strategic Planning Committee (“the Committee”). The Committee members received a stipend for participating in approximately 30–50 h of intervention activities. The changemakers also receive a stipend for their role in the intervention. The changemakers and research team met weekly to co-design the Committee meetings. After the Committee meetings concluded, the changemakers continued to meet with the research team to advance the priorities identified by the Committee. The research team provided $5,000 to LiveWell as seed funding to kick-start community-level activities that aligned with Committee priorities. The Social, Behavioral and Educational Institutional Review Board at Tufts University approved this study.

### Committee meetings

There were six Committee meetings held between October 2019 and July 2020 (four in person and two virtual). The Committee first met in October 2019, where the committee members, changemakers, and the research team introduced themselves. The central question that the Committee was exploring during meetings was “What are the factors that influence youth obesity in your community?” The group used a graph depicting stylized obesity rate trends over time in Greenville to ground the initial discussion and subsequent group model building activities. The group was encouraged to consider policy, systems, and environmental-level influences on youth obesity.

The facilitation team (changemakers and research team) led three group model building activities: “hopes and fears”, “graphs over time”, and “connection circles”. Details about these and other group model building activities, including freely available scripts for planning and facilitating group model building activities, are available elsewhere (29, 30). At the second Committee meeting in November, the group revisited trends over time and started building causal loop diagrams in small groups that showed how variables were connected in a system that influences the central problem trend of interest, childhood obesity rates in Greenville. The group continued working on causal loop diagrams in their third meeting in December. Between the third and fourth meetings, the research team and changemakers refined the diagrams by identifying similarities and differences in key variables and feedback loops across the small groups and integrating them into one causal loop diagram.

During the fourth meeting in January, the research team and changemakers presented the refined causal loop diagram back to the group and facilitated a discussion about system insights emerging from the diagram. Insights included the importance of the built environment for promoting physical activity and shaping food choices, time constraints limiting healthy food consumption, and the long-term health burdens of obesity, perpetuating high healthcare costs and limiting wealth to invest in healthy choices. Then the group discussed their priorities for where they wanted to intervene in the system depicted in the causal loop diagram. At that point the Committee decided to seek input from existing working groups from within LiveWell who were already working in areas that had surfaced during the Committee meetings, to add detail and other perspectives to the causal loop diagram.

The fifth Committee meeting was held virtually in June 2020 because of the COVID-19 pandemic. During that meeting, the Committee reviewed the causal loop diagram by subsystem, the key takeaways in each subsystem, and actions that had been generated by the group in the January meeting and mapped on to the causal loop diagram by the research team. The group then prioritized those areas considering things like scale (i.e., how many children would be reached), impact (whether LiveWell and the Committee could add value in the area), multi-sectoral contribution (whether the strategy aligns with the interests of multiple sectors), and timeline (how long it would take to see impact). Once the systems mapping was complete stakeholders participated in the “Places to Intervene” group model building activity, which uses a series of prompts to brainstorm potential solutions that could be mapped onto the systems map (31, 32). These solutions were then “ranked” by the stakeholders through group discussion and consensus on the dimensions of low to high impact and low to high feasibility, with lowest ranking interventions being those with low feasibility and low potential impact and the highest ranking being those that had high feasibility and high potential impact.

In the sixth and final Committee meeting (July 2020) the changemakers shared input from the working groups, and the research team presented a PowerPoint that highlighted evidence from peer-reviewed publications and consensus reports that aligned with the Committee’s ranked priorities. Members of LiveWell’s Leadership Team attended in addition to Committee members. The changemakers and research team facilitated the meeting, sharing an overview of the whole SDCD process; briefly summarizing scientific evidence about childhood obesity as a public health problem; highlighting key opportunities for action within the causal loop diagram the Committee created and associated evidence for why intervening on those opportunities could help improve child health; and then facilitating a discussion with the meeting attendees that resulted in approval of three action areas.

Once the GMB process was completed with the stakeholder group they were invited to join working groups around any of the areas identified as part of the GMB process in both formal (i.e., join workgroup as a formal member) or informally (i.e., provide *ad hoc* feedback and input). While stipends were no longer offered for this work, this was not a barrier to the majority of participants as most participated in the work as part of their formal employment. Beginning in 2021, the coalition instituted a process to provide compensation to community representatives who are not attending meetings as part of their employment in recognition of their time and expertise.

### Prioritized action areas

**Figure 1:**
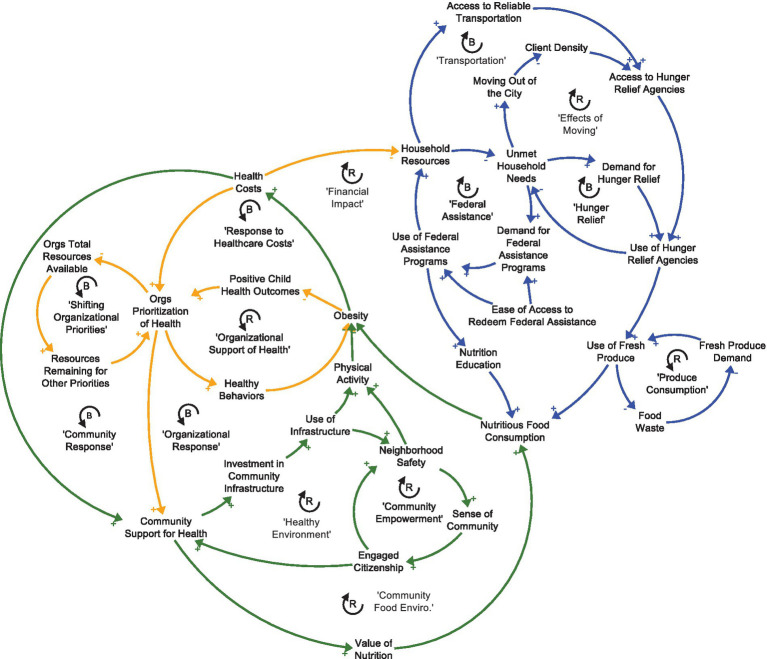
How to read a causal loop diagram: Words are variables that can increase or decrease over time. Arrows indicate that a change in the initial variable leads to a change in the variable that the arrow is point to, all else equal. Positive signs indicate that the change happens in the same direction (i.e., an increase leads to an increase, or a decrease leads to a decrease). A negative sign indicates that the change happens in the opposite direction (i.e., an increase leads to a decrease, or a decrease leads to an increase). ‘R’ represents a reinforcing feedback loop where a change in an initial variable feeds through the loop and amplifies the change in the same direction (i.e., an initial increase leads to more of an increase in the initial variable). ‘B’ represents a balancing feedback loop where a change in an initial variable feeds through the loop and switches the direction of the change in that initial variable (i.e., an initial increase leads to a decrease in the initial variable).

To date, LiveWell adopted three new action areas that helped the coalition move beyond sector and/or organization-specific change toward a larger systemic vision for whole-of-community change. Coalition staff members attributed this shift towards a more systemic vision to the SDCD intervention. The three action areas were: (1) address *food insecurity* through better utilization of federally funded nutrition programs; better coordination among food security partners; creation of the food insecurity index; and ensuring equitable access in neighborhoods experiencing the highest levels of food insecurity, (2) address *community power building* by including more community members in coalition prioritization and decision making; developing a Food Equity Action Board to expand community involvement beyond the Food Security Coalition that already existed within LiveWell; and exploring other opportunities to engage community members in coalition decision making, and (3) engage in more specific and intentional *advocacy* in government, schools, churches, businesses, early childhood and other settings to drive community engagement and build allies in changing local and state policy. Figure 1 depicts a simplified version of the Committee’s whole causal loop diagram with colors highlighting areas of the system impacted by prioritized action.

### Key insights guiding how LiveWell is addressing prioritized action areas

LiveWell has a long history of translating public health priorities into community-level work. The SDCD theory-informed intervention provided a new approach for considering the systems influencing public health trends and helping guide actions to prioritize in LiveWell’s 2021 Five-year Strategic Plan. Complimentary to the three action areas described above, the SDCD theory-informed intervention surfaced four key insights that suggested a need for a new group within LiveWell—the Food Equity Action Board—and new approaches for creating lasting change in the community. The insights aligned with several social determinants of health, including the food environment (i.e., food security, access, and availability), and political and social context (33). The insights highlight opportunities to direct community-based resources towards areas of high need and to engage in whole-of-community change, which has the potential for promoting health equity in Greenville.

### Key insight 1: participation gaps in federal nutrition program participation

Federally funded nutrition programs were not being used to their full extent (Figure 1, “Federal Assistance”). For example, there was an observed 65% drop in Special Supplemental Nutrition Program for Women, Infants, and Children (WIC) participation among eligible families after a child turns one; the Summer Meals Program was also underutilized, according to conversations with Summer Meals providers. One of the first efforts to increase use of federal nutrition programs was the initiation of a farmers’ market in partnership with a local Seventh Day Adventist Church that has a thriving urban garden in a lower resourced community in the City of Greenville. The Food Security Coalition developed marketing materials and assisted in building the infrastructure to accept Supplemental Nutrition Assistance Program (SNAP) benefits and double bucks (“Healthy Bucks”) at the market. The Coalition conducted a quality improvement project with participants to estimate demographics of farmers’ market shoppers, use of SNAP at the market, and to assess whether items sold at the market met shoppers’ needs. The research team provided additional funding beyond the intervention seed funding to support the farmers’ market survey, market gift cards as incentives for survey completion, and provided technical assistance to a graduate student who conducted and analyzed the survey.

### Key insight 2: opportunity for more coordination among food security efforts

Many of Greenville County’s food security efforts were not coordinated efficiently. While the county has many organizations working to address hunger and food insecurity, their efforts were often siloed (e.g., no infrastructure to share excess foods or communicate global community needs) or duplicative (e.g., multiple agencies serving the same food pantries, at times leading to duplicative deliveries and reducing the total number of food pantries receiving donations). Additionally, many of these services are in the city center of Greenville, which is experiencing a high rate of gentrification that is pushing existing residents out of the city center, away from these existing services (Figure 1, “Effects of Moving”, and “Transportation”). LiveWell’s Food Security Coalition helps coordinate food security services, and the coalition added detail about food insecurity to the causal loop diagram (Figure 1, “Produce Consumption”, “Effects of Moving”). LiveWell used insights from the causal loop diagram to propose infrastructure investments (e.g., cold storage) for a portion of the county’s CARES Act allocations that aimed to support the County’s hunger relief efforts in high need areas. LiveWell was awarded $1.2 million dollars of Greenville County’s CARES Act allocations in response to a collaborative, multi-partner application directed toward hunger relief and food system development; the proposal was directly informed by the CLD insights.

### Key insight 3: shift towards broad policy and systems change

There was a need to shift away from environmental-level change efforts within individual organizations towards building relationships and advocacy skills to influence broader policies and systems in the community. Examples of broader policy options include tax incentives to develop grocery stores in food insecure neighborhoods, implementing bilingual signage in parks and recreation spaces, and complete streets policies adopted by the county. The Committee agreed that maintaining strong relationships with formal and informal decision-makers and influential people should be a priority to increase community support for healthy eating and active living policy and systems change efforts. In practice, that meant the coalition would work towards building support for health-promoting policies among community members (lay leaders), elected officials, chambers of commerce, and other collaborative efforts rather than focusing on building relationships with individual schools, faith communities, or businesses (Figure 1, “Organizational Response”). Efforts with community members included developing a Food Equity Action Board and a Health Equity Action Leaders Board, composed of community lay community members with lived experience. Both groups met regularly with coalition staff and stakeholders and participated in a cohort-based learning experience about local health issues and how to advocate with their local officials about these issues. Additionally, LiveWell recently hired a Policy and Advocacy Director, with significant state and national legislative experience to work on the coalition’s behalf advocating for identified policy and systems changes at the city, county, and state levels.

### Key insight 4: citizen engagement for lasting change

A more engaged citizenship is critical to make lasting change in the community (Figure 1, “Healthy Environment”). The coalition has extensive representation from organizations that have a vested interest in changing the youth obesity rates in Greenville County. Over its history, however, LiveWell has rarely created opportunities for meaningful, long-term engagement and leadership by lay citizens. The Food Security Coalition decided that future efforts should engage community members that have experienced food insecurity, lack of physical activity opportunities, and/or obesity. As a result, the coalition has committed to building infrastructure to shift the coalition from being community-informed, meaning that the opinions of the community members are sought in defining problems and potential solutions, to becoming community-led where community members are at the center of all the work (defining problems, and co-designing solutions, and implementation). The first step of this plan was to identify and meaningfully engage 10–12 community members in a Food Equity Action Board to guide the work of the Food Security Coalition. To date, a Food Security Community Mobilizer has been hired to facilitate and support the Food Equity Action Board, the initial group of board members have been selected, and the group has begun meeting. In addition to seed funding from Catalyzing Communities, LWG leveraged local foundation and federal grant dollars to support the following implementation activities: start the board by hiring a community member to consult on the planning efforts to ensure diversity and cultural appropriateness; provide stipends for Board participants; and implement efforts prioritized by the group.

### Building capacity to use community-based system dynamics in Greenville

**Figure 2:**
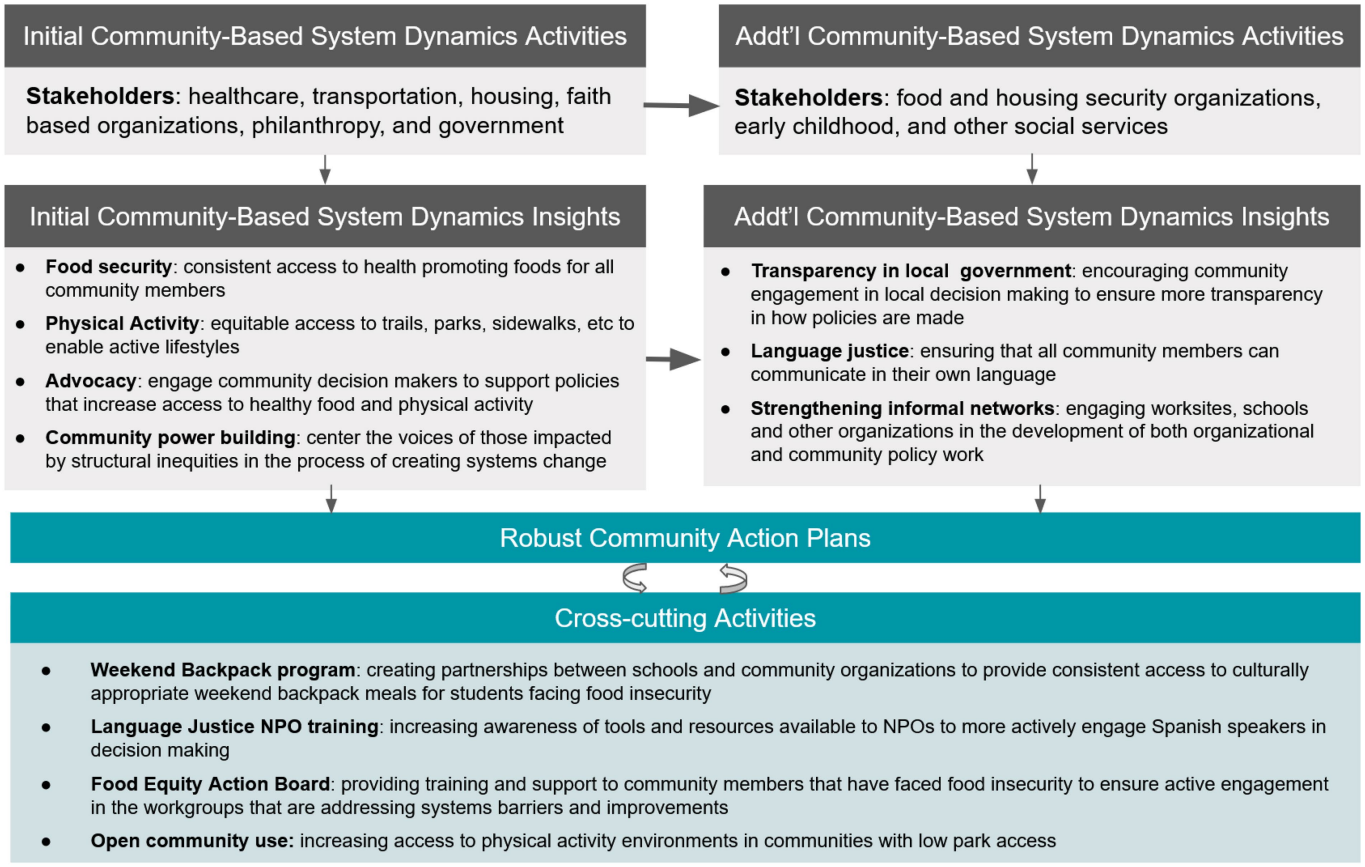
Diagram of actions, groups, and use of group model building stemming from a Stakeholder-driven Community Diffusion-informed intervention implemented with LiveWell Greenville (2019 – 2021).

A core principle of CBSD is to build communities’ capacity to use group model building and systems dynamics concepts (24). This principle was realized, as demonstrated by the uptake and use of group model building for understanding systems by other LiveWell-supported initiatives beyond the initial activities in the SDCD intervention. The CBSD approach was adapted to assist in coalition building and planning efforts for several other active workgroups including: LiveWell Early Childhood; LiveWell at Worship’s Race Relations Subcommittee; and the Build Trust, Build Health Initiative. For example, LiveWell at Worship’s Race Relations Subcommittee used group model building to explore drivers of racial inequities in Greenville, including economic and education inequities. The process helped the multi-racial and multi-ethnic group share their perspectives, and at times deeply personal experiences, promoting a holistic view of many of the drivers and consequences of racial inequities in the community. The Subcommittee is using this understanding to advance racial equity in their community by engaging in advocacy for more transparency in local politics, state level policies to increase access to SNAP, and advocacy for grocery stores with a pharmacy in several neighborhoods. In addition, LiveWell’s Executive Director has provided technical assistance to other Greenville County coalitions wishing to employ group model building and CBSD principles in their work. Figure 2 shows the uptake of CBSD-informed projects in LiveWell, starting with the Stakeholder-driven Community Diffusion-informed intervention and spreading beyond the topic of childhood obesity to other important topics. LiveWell has a long history of using extensive community action plans to guide its work; however, the CBSD process brought a new level of sophistication to the plans, resulting in rapid and meaningful changes.

## Discussion

Since its inception, LiveWell has focused on PSE change approaches to support healthy eating and active living changes in Greenville County. Historically, much of this work focused on organizational policy change within sectors such as schools, work, out of school time, and faith-based settings. In 2019, LiveWell recognized the need to focus on community-level factors to effectively drive whole-of-community change. CBSD, an established approach employed in the SDCD intervention, provided an avenue to develop a collective and comprehensive understanding of the local contextual factors that were driving community-level obesity prevalence and its associated disparities. The approach also helped LiveWell gain traction to address food insecurity, particularly as the COVID19 pandemic further strengthened the need for better communication and collaboration among food security partners with a sense of urgency that was not felt in the past. This was due in part to the fact that food insecurity emerged as such a central component of the causal loop diagram that they developed together over the course of the intervention. The insights and priorities that surfaced during the intervention provided a strong foundation to seek funding that became available quickly through COVID-related relief funds. LiveWell was awarded $1.2 million in CARES Act funding that was immediately allocated toward priority areas identified during the SDCD intervention, as well as other priority areas identified by the coalition. Moreover, the novel approaches helped LiveWell establish new partnerships and supported multi-sector partners in seeing their role in a complex system that affects health and well-being for all Greenville community members.

CBSD, group model building, and systems thinking have successfully been incorporated into other child obesity prevention interventions. In Australia, the GenR8 Change approach included group model building with over 100 community leaders and members who then committed to join working groups to plan and implement obesity prevention actions such as creating sugar free zones and promoting breastfeeding (34). The SDCD-informed intervention took a somewhat different approach than GenR8 by spending more time with the smaller multi-sector group in Greenville as they developed systems maps and insights. Partnering with a local “backbone” organization was similar in both studies (35). The same research group is conducting a cluster randomized control trial to test whether a participatory systems science intervention can strengthen community action for obesity prevention and whether actions can decrease risk factors for child obesity (36). In the US, the National Academies of Science’s Roundtable on Obesity Solutions used group model building to inform a strategic plan that researchers, organizations, and institutions can refer to as they engage in obesity prevention and treatment efforts (37). Another intervention created a systems science curriculum to engage African American youth in exploring environmental factors that influence obesity (38). Study findings suggest the approach may have changed youth’s perceptions about important drivers of obesity and lead to an increase in youth’s support for policy changes that promote healthier food environments (38). As the field of public health continues to emphasize the need to use systems thinking to address complex challenges, more applications of approaches like those used in the SDCD-informed intervention are likely to emerge (39–41).

### Lessons learned for future applications of stakeholder-driven community diffusion-informed interventions

The insights that came out of the LiveWell—Catalyzing Communities partnership were important both in how they impacted follow-on work with LWG, and in how they helped shape future implementation of the SDCD-intervention in five communities that have since joined the Catalyzing Communities project.

#### Committee participation

The changemakers and research team learned that while selecting high-level leadership might mean they are powerful within their organization, it also meant that they might be removed from the day-to-day work of the coalition and their partners, and thus were less involved in the implementation of priorities identified through the intervention. If the coalition implemented the process again, it would choose a broader mix of stakeholders, ranging from community members to organizational leaders and decision-makers, that would continue to be engaged in implementing actions prioritized in the SDCD intervention. This shift is reflective of a broader change in implementation of the SDCD intervention in other communities engaged in the Catalyzing Communities project, as a way of bringing together voices that can inform a holistic view of a complex issue, valuing lived experience, empowering participants, and supporting relationship- and network-building across sectors and across the community.

#### Virtual engagement

COVID-19 made sustaining engagement of the group difficult, and the coalition had to transition to virtual platforms that required intentional planning and creative virtual solutions to facilitate the group model building process (42). The virtual resources developed as part of this process were integral to the group’s success and have been employed by the coalition across numerous workgroups to facilitate meaningful dialogue and action planning when in-person meetings were not possible.

#### Value of systems insights to expand and enhance coalition efforts

One of the challenges in engaging the Committee in developing the causal loop diagram is that some of the issues and root causes associated with obesity in the community were outside LiveWell’s scope of expertise and mission. The changemakers and research team learned that in the action planning process they had to balance the Committee’s focus on PSE change while also pushing the boundaries of the coalition to adopt health equity and social determinants of health approaches in the coalition’s work.

The causal loop diagram helped participants from sectors that were more distal to obesity prevention, like the housing and redevelopment authority and houses of worship, see the important role they played and the expertise they brought to discussions. It also allowed them to see where there was synergy across the partners’ work that extended beyond working with LiveWell. By using systems-thinking, participants realized that they did not have to act on every component of the system to drive significant change in obesity prevention; rather, if they could impact one component of a causal loop it could create a cascade effect that could subsequently impact other aspects of the system. It also allowed the Committee and the coalition to visualize where there was the greatest shared interest and available resources across partner organizations to prioritize action that could result in measurable change. Finally, the portfolio of structured group model building activities facilitated participation and meaningful input across all committee members, which has potential to better balance power dynamics compared to traditional strategic planning approaches if implemented in other places.

### Limitations

LiveWell is an established non-profit coalition with 10 years of successes that contribute to its credibility and leadership role in the Greenville community. The coalition currently has seven paid staff who support coalition activities, initiatives, and fund-raising, as well as strong partnerships with Furman University whose faculty, staff, and students provide support for evaluation and community engagement. Given its financial resources and social capital, LiveWell was well positioned to implement and benefit from an SDCD-informed intervention; other coalitions may not be as well-positioned. Another limitation is that this is a case study; we did not rigorously test the effect of the SDCD intervention in a controlled trial with a comparison group. The detail provided in this case study, however, may be useful to groups trying to implement coalition interventions to change systems in their communities.

A large component of the SDCD intervention relies on CBSD principles and group model building skills. Building the pool of individuals with such skills through formal training mechanisms is crucial for scaling up SDCD interventions and similar interventions. The intervention featured in this case study did not employ quantitative simulation modeling that is argued by some in the system dynamics field to be crucial for developing rigorous system insights and determining effective policies (43). Others in the field recognize the utility of informal and qualitative models, like the causal loop diagram developed in this intervention, for helping groups solve learning and coordination problems (44, 45). Quantitative system dynamics simulation models are appropriate when the purpose of the model is for rigorous analysis of objective policies within an existing system and well suited for solving analytical problems when sufficient resources are available to build and test such models. Qualitative models can generate actionable systems insights and, importantly, promote learning and build consensus among participating individuals to initiate and sustain actions (46, 47). Now that the LiveWell coalition has developed some CBSD capabilities and the purpose of modeling necessitates additional insights from simulation, they are working with the research team to explore opportunities to develop quantitative system dynamics models.

## Conclusion

This case study describes a theory-informed intervention process to intervene with a group of multi-sector stakeholders to promote whole-of-community change. The process led to three new priority areas for the coalition: (1) addressing food insecurity; (2) a focus on building power within the community; and (3) new advocacy goals to promote systems change beyond the coalition’s previous focus on PSE change within individual organizations and sectors. The intervention prompted the application of CBSD to other topics beyond childhood obesity, which could lead to paradigm shifts about how to address complex, entrenched public health issues in the community.

## Data availability statement

The original contributions presented in the study are included in the article/supplementary material, further inquiries can be directed to the corresponding author.

## Ethics statement

The studies involving human participants were reviewed and approved by Tufts University IRB. The patients/participants provided their written informed consent to participate in this study.

## Author contributions

LC, KW, SW, MF, and JA helped to implement the intervention and contributed to the analysis and manuscript writing. SW is the Executive Director of LiveWell Greenville and MF is the Principal Investigator for LiveWell at Furman University. EH and TM contributed to analysis, interpretation, and manuscript writing. CE conceptualized the study and obtained funding. All authors contributed to the article and approved the submitted version.

## Funding

This research was funded by the JPB Foundation.

## Conflict of interest

The authors declare that the research was conducted in the absence of any commercial or financial relationships that could be construed as a potential conflict of interest.

## Publisher’s note

All claims expressed in this article are solely those of the authors and do not necessarily represent those of their affiliated organizations, or those of the publisher, the editors and the reviewers. Any product that may be evaluated in this article, or claim that may be made by its manufacturer, is not guaranteed or endorsed by the publisher.
